# Factors predictive of hospital admission for children via emergency departments in Australia and Sweden: an observational cross-sectional study

**DOI:** 10.1186/s12913-023-09403-w

**Published:** 2024-02-23

**Authors:** Julia Crilly, Amy Sweeny, Åsa Muntlin, David Green, Lorelle Malyon, Luke Christofis, Malcolm Higgins, Ann-Sofie Källberg, Sara Dellner, Åsa Myrelid, Therese Djärv, Katarina E. Göransson

**Affiliations:** 1 Department of Emergency Medicine, Gold Coast Health, 1 Hospital Blvd, Southport, QLD 4215 Australia; 2 School of Nursing and Midwifery, Griffith University, Southport, QLD Australia; 3 Department of Medical Sciences/Clinical Epidemiology, Uppsala University, Uppsala, Sweden; 4 Department of Public Health and Caring Sciences/Health Services Research, Uppsala University, Uppsala, Sweden; 5 Emergency Department, Queensland Children’s Hospital, Children’s Health Queensland, Brisbane, QLD Australia; 6 Emergency Department, Lyell McEwin Hospital, Elizabeth Vale, South Australia, Australia; 7 Paediatric Emergency Department, Women’s and Children’s Hospital, North Adelaide, South Australia Australia; 8 School of Health and Welfare, Dalarna University, Falun, Sweden; 9 Department of Emergency Medicine, Falun Hospital, Falun, Sweden; 10 Maternal Health Care Unit, Region Stockholm, Stockholm, Sweden; 11 Department of Women’s and Children’s Health, Uppsala University Children’s Hospital, Uppsala, Sweden; 12 Emergency and Reparative Medicine Theme, Karolinska University Hospital, Stockholm, Sweden; 13 Department of Medicine, Karolinska Institutet, Solna, Stockholm, Sweden

**Keywords:** Emergency departments, Children, Hospital admission, Australia, Sweden

## Abstract

**Background:**

Identifying factors predictive of hospital admission can be useful to prospectively inform bed management and patient flow strategies and decrease emergency department (ED) crowding. It is largely unknown if admission rate or factors predictive of admission vary based on the population to which the ED served (i.e., children only, or both adults and children). This study aimed to describe the profile and identify factors predictive of hospital admission for children who presented to four EDs in Australia and one ED in Sweden.

**Methods:**

A multi-site observational cross-sectional study using routinely collected data pertaining to ED presentations made by children < 18 years of age between July 1, 2011 and October 31, 2012. Univariate and multivariate analysis were undertaken to determine factors predictive of hospital admission.

**Results:**

Of the 151,647 ED presentations made during the study period, 22% resulted in hospital admission. Admission rate varied by site; the children’s EDs in Australia had higher admission rates (South Australia: 26%, Queensland: 23%) than the mixed (adult and children’s) EDs (South Australia: 13%, Queensland: 17%, Sweden: 18%). Factors most predictive of hospital admission for children, after controlling for triage category, included hospital type (children’s only) adjusted odds ratio (aOR):2.3 (95%CI: 2.2–2.4), arrival by ambulance aOR:2.8 (95%CI: 2.7–2.9), referral from primary health aOR:1.5 (95%CI: 1.4–1.6) and presentation with a respiratory or gastrointestinal condition (aOR:2.6, 95%CI: 2.5–2.8 and aOR:1.5, 95%CI: 1.4–1.6, respectively). Predictors were similar when each site was considered separately.

**Conclusions:**

Although the characteristics of children varied by site, factors predictive of hospital admission were mostly similar. The awareness of these factors predicting the need for hospital admission can support the development of clinical pathways.

**Supplementary Information:**

The online version contains supplementary material available at 10.1186/s12913-023-09403-w.

## Introduction

The overall number of patient presentations made to emergency departments (EDs) have increased over the years in many developed countries, including Sweden [1, 2], Australia [3], the United States (US) [4], and England [5]. This growth includes children, where the number of ED presentations made to public hospitals have increased from 1.4 M in 2011–12 to 1.7 M in 2021–22 (for those aged ≤ 14 years) in Australia [3, 6], from 3.5 M in 2011–12 to 4.7 M in 2021–22 (for those aged ≤ 14 years) in England [5, 7] and from 32.1 M in 2016 to 34.7 M in 2019 (for those aged < 18 years) in the US [8]. In Australia, children ≤ 14 years of age comprised 19.5% of the 8.8 million presentations made to public hospital EDs in 2021–22 [3]; in England, children ≤ 14 years of age comprised 20.5% of the 22.8 M ED presentations in 2021–22 [5], and in the US, children < 18 years of age comprised 23.1% of the 150.6 M ED presentations in 2019 [8].

A commonly reported measure in emergency health services research is admission rate. Wide variation in the reporting of childhood admission rate is however evident. For example, in Australia and New Zealand, the admission rate from EDs for children aged ≤ 18 years was 24% [9], in Sweden, the admission rate for children aged ≤ 17 years was 19% [10], in the Netherlands, the admission rate from children aged < 16 years was 20–23%, and in England, the admission rate from children aged < 16 years was 10% [11]. Being able to predict if a child is likely to require admission early in the ED journey may be useful to prospectively inform bed management and patient flow strategies, speed up the admission process, and decrease ED crowding.

Models predicting admission from ED have been developed in several countries [12]. From studies undertaken in Australia, factors associated with hospital admission for adults have been reported to include older age, arrival by ambulance, higher triage urgency classification [13, 14], referral from local doctor, need for blood test, arrival during evening hours, weekday presentation [13], previous admission (within 30 days), and presenting problem (gastrointestinal, febrile illness, social) [14]. From studies undertaken in the US and Ireland, factors associated with hospital admission for children have been reported and include: various presenting problems [15–20], weekday presentation [18], triage classification [15–18], arrival by ambulance [16, 19], distance travelled [17, 18], and abnormal vital signs [15–17, 19, 20]. It remains largely unknown though, if admission rate or other factors are predictive of admission for children based on the population to which the ED served (i.e., children only, or both adults and children) and in other countries. Examining variation in these aspects between hospitals domestically and internationally allows opportunities for shared learning and practice and process development as well as enhanced research understanding. The use of routinely collected data to inform admission likelihood is one way to assist this process.

The aim of this study was to describe the profile of and identify factors predictive of hospital admission for children who presented to four EDs in Australia and one ED in Sweden as some similar, but also different demographic profiles and health system structures exist between these two countries. Life expectancy and common causes of death (heart disease, Alzheimer’s disease, chronic obstructive pulmonary disease) are similar in both countries [21]. Issues with ED crowding [22, 23] and pressures to meet performance targets are also common across both countries [24, 25]. Despite these similarities, differences exist in terms of the percentage of population with private health insurance (less frequently utilised in children): 5% in Sweden and 50% in Australia [26], and the specialisation of emergency medicine which is much newer in Sweden, compared to Australia.

## Methods

### Study design, setting and sample

This was a multi-site observational cross-sectional study conducted using routinely collected data from five hospital EDs: two in Queensland (QLD), two in South Australia (SA), and one in Sweden to offer some international comparability. The sites were specifically chosen for their variation in terms of setting and servicing population. Table 1 provides an overview of each study site. We included data from all patient presentations made to the EDs by children < 18 years of age between July 1, 2011 and October 31, 2012.

**Table 1:** Overview of study sites

**Site Characteristic** **(based on 2011)**	**Hospital A South Australia**	**Hospital B Queensland**	**Hospital C** **Queensland**	**Hospital D** **South Australia**	**Hospital E** **Middle Sweden**
**Population served**	Children	Children	Children and adults	Children and adults	Children and adults
**Hospital type**	Public, teaching, tertiary	Public, teaching, metropolitan	Public, teaching, regional	Public, teaching, metropolitan	Public, teaching, metropolitan
**Annual ED presentations**	44,000	29,000	65,000	50,000	60,000
**Admission rate**	27%	22%	25%	31%	33%
**% Children (<18 years)**	100%	100%	20%	25%	23%
**Staffing profile:**					
**No. FTE Nurses (all types)**	80	42	120	110	109
**No. FTE Medical (all types)**	28	26	73	75	70
**Observation ward / Short Stay Unit**	Yes	Yes	Yes	Yes	Yes
**Observation ward / Short Stay Unit caters for children**	Yes	Yes	No	Yes	No
**Fast track**	Yes	Yes	Yes	Yes	Yes
**Nurse Practitioners**	NP (in training)	No	Yes	Yes	No^a^

*ED* Emergency department, *FTE* Full-time equivalent

^a^ Emergency Nurse practitioners did not exist in Sweden at the time of the study

### Data collection

Data used for this study was based on routinely collected demographic and clinical data obtainable from each site’s ED information system. Variables requested were informed by those reported in previous research (e.g., triage category, mode of arrival, presenting problem) [15, 16, 20], and expert clinical advice. In preparation for analysis data provided required standardisation and were categorised and coded (Supplementary Material, Table S1). For four variables (triage category, referral source, discharge disposition, and arrival mode) consultation with investigators at each site was required to support standardisation. Hospital admission in our study was defined as an admission to the study site hospital (either short stay unit, hospital-in-the-home, or non-emergency department hospital ward), aligning with national reporting [3].

### Data analysis

Descriptive statistics were used to describe demographic characteristics, ED clinical characteristics and outcomes for all presentations. Median and inter-quartile range (IQR) were used for continuous variables that were not normally distributed. Frequency distributions were used for categorical variables. Inferential statistics were used to test for differences between groups, including the chi-square test for categorical variables and the independent samples median test for comparisons of medians.

Variables with more than 10% missing data were excluded from models predicting admission. The amount of missing data by site, and for all sites combined, is presented in Supplementary Material, Table S2. Custom models were built for each site individually (due to varied data availability). Crude odds ratios (OR) and 95% confidence intervals (CI) were produced for each variable that had 95% or more of data complete at each site, and multivariate regression was performed for each site, producing Adjusted OR (aOR).

One multi-site multivariate model was developed including all sites’ data, and testing the variables age category, sex, hospital type (children’s or mixed), season, time of arrival, day type of arrival (weekday or weekend), arrival mode, referral source, triage category and the top five major diagnostic categories. Analysis of the variables: Aboriginal and Torres Strait Islander status, insurance status, and language spoken, was limited to crude ORs and 95% CIs at the multi-site level because these were collected at Australian sites only. Multi-variate models were also developed for each site.

Multivariate regression was performed using a forward conditional approach. A two-sided *p*-value of < 0.05 was considered statistically significant. Data were analysed using SPSS (IBM Corp. SPSS Version 22.0. Armonk, NY).

The study was approved by the Health Services (QLD: HREC/13/QPAH/347; SA: HREC/14/WCHN/66) and Griffith University (NRS/05/15/HREC) Human Research Ethics Committees in Australia as well as the Swedish Regional Ethical Board in Sweden (Dnr: 2013/11636–31-/1; 2014/566–32).

## Results

### Demographic characteristics

A total of 151,647 ED presentations were made to the five EDs over the 15-month period by children aged under 18 years. The demographic characteristics of children are presented in Table 2. Whilst the median age was 4 years (IQR 1–11) for all sites combined, children presenting to the three mixed-EDs tended to be slightly older. The proportion of children in the oldest age group (12–17 years) varied by site, from 11.6% of presentations at the children’s hospital in QLD to 31.2% at the mixed hospital in QLD. A higher proportion of males presented to all EDs (55%, n = 83,227). This was consistent across sites. At the Australian sites, the proportion of presentations from children of Aboriginal or Torres Strait Islander ethnicity ranged from 2.7% in the QLD mixed ED to 7.0% in the SA mixed ED.

**Table 2:** Characteristics of children presenting at five hospital EDs in Australia and Sweden

	**ED A, SA [Children’s]**		**ED B, QLD [Children’s]**		**ED C, QLD [Mixed]**		**ED D, SA [Mixed]**		**ED E, Sweden [Mixed]**		**Total**	
	n	%	n	%	n	%	n	%	n	%	n	%
**Age category**												
< 12 months	9315	15.6	6096	16.2	2482	13.2	3352	17.2	2416	15.0	23661	15.6
1	8219	13.8	5560	14.8	2122	11.3	2444	12.5	1844	11.4	20189	13.3
2-5	16547	27.7	12264	32.6	4455	23.8	5082	26.1	3685	22.9	42033	27.7
6-11	12695	21.3	9302	24.8	3835	20.5	3684	18.9	3545	22.0	33061	21.8
12-17	12961	21.7	4355	11.6	5847	31.2	4924	25.3	4616	28.7	32703	21.6
**Median age (IQR) years**	4 [1.0-11.0]		3 [1.0-8.0]		6 [2.0-13.0]		4 [1.0-12.0]		6 [1.0-12.0]		4 [1.0-11.0]	
**Sex**												
Male	32655	54.7	21215	56.5	10271	54.8	10590	54.3	8496	52.8	83227	54.9
Female	27082	45.3	16361	43.5	8467	45.2	8896	45.7	7610	47.2	68416	45.1
**Indigenous status**												
Not Aboriginal or Torres Strait Islander	57333	96.0	35852	95.4	17891	95.5	17920	92.0	Not available		128996	95.2
Aboriginal or Torres Strait Islander (any)	2257	3.8	1300	3.5	498	2.7	1364	7.0			5419	4.0
Missing	147	0.2	424	1.1	349	1.9	202	1.0			1122	0.8
**Language spoken at home**												
English	13064	21.9	36437	97.0	18291	97.6	18281	93.8	Not available		86073	63.5
Not English	26	0.0	960	2.6	202	1.1	267	1.4			1455	1.1
Missing	46647	78.1	179	0.5	245	1.3	938	4.8			48009	35.4
**Insurance status**												
Medicare only	34680	58.1	22067	58.7	17719	94.6	17196	88.2	Not available		91662	67.6
Private Health	24906	41.7	15228	40.5	711	3.8	2049	10.5			42894	31.6
No insurance	0	0.0	81	0.2	94	0.5	0	0.0			175	0.1

*ED* Emergency department, *SA* South Australia, *QLD* Queensland. *IQR* Inter-quartile range

### ED characteristics

The ED characteristics of children are presented in Table 3. Across all sites, most children (87%, *n* = 131,698) arrived to the ED through privately arranged transport, with 13% (*n* = 19,285) arriving by ambulance, although this ranged from 7% in the Swedish mixed ED to 22% in the QLD mixed ED. Considerable variation in assigned triage categories between sites was noted, with 7.4% of presentations to the SA mixed ED considered emergency (requiring attention immediately/within 10 min) and 20.6% assigned this urgency at the Swedish ED. The proportion of children presenting on the weekend (29.7%) or after-hours (43.8%) was relatively consistent across sites. Across all sites, the top five most common ED assigned diagnostic categories were trauma (26%, *n* = 38,629), infectious disease (14%, *n* = 20,971), ear, nose and throat (ENT) condition (9%, *n* = 13,162), gastrointestinal condition (8%, *n* = 12,636), and respiratory condition (8%, *n* = 11,639). The proportion of trauma presentations was higher at the mixed EDs when compared to the children’s EDs.

**Table 3:** Emergency department characteristics of children presenting to five hospitals in Australia and Sweden

	**ED A, SA [Children's]**		**ED B, QLD [Children's]**		**ED C, QLD [Mixed]**		**ED D, SA [Mixed]**		**ED E, Sweden [Mixed]**		**All hospitals [n=151647]**	
	n	%	n	%	n	%	n	%	n	%	n	%
**Referral source**												
Self	51809	86.7	31466	83.7	16493	88.0	17121	87.9	14241	88.4	131130	86.5
Primary health	5732	9.6	3539	9.4	1781	9.5	1894	9.7	1405	8.7	14351	9.5
Transfer-in	1866	3.1	1529	4.1	236	1.3	39	0.2	1	0.0	3671	2.4
Planned return	50	0.1	869	2.3	51	0.3	100	0.5	453	2.8	1523	1.0
Community	173	0.3	81	0.2	137	0.7	79	0.4	0	0.0	470	0.3
Missing	107	0.2	92	0.2	40	0.2	253	1.3	6	0.0	498	0.3
**Arrival mode**												
Private transport	52930	88.6	31815	84.7	14453	77.1	17593	90.3	14907	92.6	131698	86.8
Ambulance	6634	11.1	5699	15.2	4178	22.3	1671	8.6	1103	6.8	19285	12.7
Other	171	0.3	63	0.2	110	0.6	65	0.3	96	0.6	505	0.3
**Triage Category**												
Immediate	86	0.1	252	0.7	134	0.7	128	0.7	419	2.6	1019	0.7
Within 10 min	6224	10.4	4205	11.2	2522	13.5	1299	6.7	2898	18.0	17148	11.3
Within 30 min	18812	31.5	12222	32.5	11897	63.5	8449	43.4	NA		51380	33.9
Within 60 min	31858	53.3	17377	46.2	3861	20.6	7121	36.5	7132	44.3	67349	44.4
Within 120 min	2757	4.6	3521	9.4	327	1.7	2489	12.8	3548	22.0	12642	8.3
Within 240 min	NA		NA		NA		NA		11	0.1	11	0.0
Missing	0	0.0	0	0.0	0.0	0.0	0.0	0	2098	13.0	2098	1.4
**Departure status**												
Home	42110	70.5	28516	75.9	13118	70.0	14322	73.5	13129	81.5	111195	73.3
Admitted	15583	26.1	8606	22.9	3147	16.8	2578	13.2	2969	18.4	32883	21.7
Did not wait	1769	3.0	343	0.9	2303	12.3	1855	9.5	0	0.0	6270	4.1
Deceased	3	0.0	4	0.0	7	0.0	1	0.0	3	0.0	18	0.0
Unknown	272	0.5	107	0.3	166	0.9	730	3.7	5	0.0	1280	0.8
**Day type of arrival**												
Weekday	42460	71.1	26094	69.4	12912	68.9	13808	70.9	11369	70.6	106643	70.3
Weekend	17277	28.9	11483	30.6	5829	31.1	5678	29.1	4737	29.4	45004	29.7
**Time of arrival** ^a^												
In-hours	34472	57.7	22731	60.5	10552	56.3	10166	52.2	9247	57.4	87168	57.5
After-hours	25265	42.3	14846	39.5	8189	43.7	9320	47.8	6859	42.6	66479	43.8
**Season of arrival**												
Spring	18771	31.4	11025	29.3	6105	32.6	6147	31.5	3618	22.5	45666	30.1
Summer	10063	16.8	6176	16.4	3146	16.8	3216	16.5	4456	27.7	27057	17.8
Autumn	19979	33.4	12746	33.9	5917	31.6	6462	33.2	3177	19.7	48413	31.9
Winter	10924	18.3	7630	20.3	3573	19.1	3661	18.8	4855	30.1	30719	20.3
**Major Diagnostic Category**												
Trauma	14505	24.3	9074	24.2	5977	31.9	5235	28.3	3838	35.6	38629	25.5
Infectious	8231	13.8	6510	17.3	1791	9.6	2715	14.7	1724	16.0	20971	13.8
Ear, nose, throat	5291	8.9	4281	11.4	1459	7.8	1475	8.0	656	6.1	13162	8.7
Gastrointestinal	6331	10.6	2208	5.9	953	5.1	1492	8.1	1652	15.3	12636	8.3
Respiratory	5220	8.7	2923	7.8	1181	6.3	1757	9.5	558	5.2	11639	7.7
Miscellaneous	2470	4.1	1910	5.1	2511	13.4	971	5.2	558	5.2	8420	5.6
Symptoms	2341	3.9	1437	3.8	629	3.4	652	3.5	391	3.6	5450	3.6
Dermatology	1693	2.8	1010	2.7	351	1.9	433	2.3	181	1.7	3668	2.4
Neurological	1389	2.3	956	2.5	398	2.1	408	2.2	370	3.4	3521	2.3
Orthopaedic	1272	2.1	606	1.6	328	1.8	394	2.1	202	1.9	2802	1.8
Psychiatric	1672	2.8	287	0.8	333	1.8	224	1.2	64	0.6	2580	1.7
Obstetrics/ Gynaecology	1144	1.9	84	0.2	164	0.9	457	2.5	29	0.3	1878	1.2
Renal	769	1.3	515	1.4	248	1.3	220	1.2	53	0.5	1805	1.2
Environmental	567	0.9	659	1.8	295	1.6	204	1.1	34	0.3	1759	1.2
Toxicology	874	1.5	221	0.6	255	1.4	251	1.4	47	0.4	1648	1.1
Urology	679	1.1	317	0.8	162	0.9	117	0.6	159	1.5	1434	0.9
Iatrogenic	531	0.9	696	1.9	132	0.7	66	0.4	4	0.0	1429	0.9
Ophthalmology	529	0.9	344	0.9	133	0.7	123	0.7	22	0.2	1151	0.8
Cardiovascular	597	1.0	120	0.3	123	0.7	214	1.2	54	0.5	1108	0.7
Haematology	271	0.5	433	1.2	62	0.3	22	0.1	0	0.0	788	0.5
Endocrine	344	0.6	143	0.4	67	0.4	66	0.4	12	0.1	632	0.4
Metabolic	192	0.3	114	0.3	64	0.3	38	0.2	10	0.1	418	0.3
Immunological	65	0.1	13	0.0	7	0.0	8	0.0	42	0.4	135	0.1
Neoplasia	45	0.1	31	0.1	4	0.0	7	0.0	24	0.2	111	0.1
Missing	2715	4.5	2685	7.1	1114	5.9	1937	10.5	5422	50.3	13873	9.1

^a^Time of arrival: In-hours: 06:00-17:59; After-hours: 18:00-05:59.

*ED* Emergency department, *SA* South Australia, *QLD* Queensland, *NA* Not available

About 22% of children were admitted to hospital following ED presentation; this varied between sites, from 13.2% to 26.1% at the SA mixed and SA children’s hospitals, respectively. The admission rate for children at both children’s EDs was higher (23% and 26%) than the three mixed EDs (13%-18%), with an increased likelihood of admission at children’s hospitals (crudeOR 1.5, 95% CI: 1.5–1.5). For the Australian sites combined, 6,270 children (4.6%) did not wait to see a doctor in the time period, although this ranged considerably between sites (0.9%-12.3% at the QLD children’s and mixed EDs, respectively).

### Predictors of hospital admission

In univariate regression analysis, except for sex, all variables tested were statistically significantly associated with hospital admission. Compared to the Swedish model, children presenting to any of the Australian sites except for the SA mixed hospital, had a higher likelihood of admission. For the Australian sites, children of Aboriginal or Torres Strait Islander ethnicity were more likely to be admitted than their non-Indigenous counterparts (OR 1.3, 95% CI: 1.2–1.4), whilst not having insurance and not speaking English were significantly associated with a lower likelihood of admission (Table 4).

**Table 4:** Predictors of hospital admission for all sites combined for children under 18 years of age

Predictor	Discharged (*n=*108,997) (78.6%)		Admitted (*n=*29,628) (21.4%)		Crude OR & 95% CI	Adjusted OR & 95% CI
	n	%	n	%		
**Hospital ED**						
SA children’s	41620	74.7	14077	25.3	1.6 (1.5-1.6)	Not tested
QLD children’s	27354	78.9	7303	21.1	1.2 (1.2-1.3)	
QLD mixed	12994	81.4	2962	18.6	1.1 (1.0-1.1)	
SA mixed	14160	84.9	2517	15.1	0.8 (0.8-0.9)	
Swedish mixed	12869	82.3	2769	17.7	1.0 (Reference)	
**Hospital ED type**						
Children’s	68974	76.3	21380	23.7	1.5 (1.5-1.5)	2.3 (2.2-2.4)
Mixed	40023	82.9	8248	17.1	1.0 (Reference)	1.0 (Reference)
**Location**						
South Australia	55780	77.1	16594	22.9	1.4 (1.3-1.4)	Not tested
Queensland	40348	79.7	10265	20.3	1.2 (1.1-1.2)	
Sweden	12869	82.3	2769	17.7	1.0 (Reference)	
**Age category**						
<12 months	16290	74.8	5487	25.2	1.1 (1.1-1.2)	0.9 (0.9-0.9)
1	14744	79.4	3814	20.6	0.9 (0.8-0.9)	0.8 (0.8-0.9)
2-5	30855	80.1	7679	19.9	0.8 (0.8-0.9)	0.8 (0.8-0.9)
6-11	24514	80.6	5903	19.4	0.8 (0.8-0.8)	0.8 (0.8-0.8)
12-17	22594	77.0	6745	23.0	1.0 (Reference)	1.0 (Reference)
**Median age (IQR) years**	4 [1,10]		4 [1,10]		4 [1,11]	
**Sex**						
Male	59774	78.5	16413	21.5	1.0 (Reference)	Did not enter
Female	49221	78.8	13213	21.2	1.0 (1.0-1.0)	
**Indigenous status**						
Aboriginal or Torres Strait Islander	3471	73.9	1224	26.1	1.3 (1.2-1.4)	Not tested
Not Aboriginal or Torres Strait Islander	92023	78.3	25457	21.7	1.0 (Reference)	
*missing*	*13503*	*12.4*	*2947*	*9.9*		
**Language spoken at home**						
English	63128	81.6	14246	18.4	1.0 (Reference)	Not tested
Not English	1124	84.3	210	15.7	0.8 (0.7-1.0)	
*missing*	*44745*	*41.1*	*15172*	*51.2*		
**Insurance status**						
Medicare only	64353	78.4	17767	21.6	1.0 (Reference)	Not tested
Private Health	31257	77.7	8956	22.3	1.0 (1.0-1.1)	
No insurance	125	85.6	21	14.4	0.6 (0.4-1.0)	
*missing*	*13262*	*12.2*	*2884*	*9.7*		
**Referral source**						
Self	98554	79.3	25745	20.7	1.0 (Reference)	1.0 (Reference)
Primary health	10120	72.7	3800	27.3	1.4 (1.4-1.5)	1.5 (1.4-1.6)
Community	323	79.6	83	20.4	1.0 (0.8-1.3)	-----
**Arrival mode**						
Private transport	99284	81.5	22604	18.5	1.0 (Reference)	1.0 (Reference)
Ambulance	9407	57.7	6907	42.3	3.2 (3.1-3.3)	2.8 (2.7-2.9)
Other	306	72.3	117	27.7	1.7 (1.3-2.1)	-----
**Triage Category** ^a^						
Emergency	7738	45.4	9308	54.6	21.4 (19.6-23.3)	20.8 (18.6-23.3)
Urgent	88699	82.0	19521	18.0	3.9 (3.6-4.2)	4.8 (4.3-5.3)
Less urgent	10745	94.7	605	5.3	1.0 (Reference)	1.0 (Reference)
*missing*	*1815*	*1.7*	*194*	*0.7*		
**Day type of arrival**						
Weekday	76022	78.0	21460	22.0	1.0 (Reference)	1.0 (Reference)
Weekend	32975	80.1	8168	19.9	0.9 (0.9-0.9)	0.9 (0.9-0.9)
**Time of arrival** ^b^						
In-hours	63640	78.3	17686	21.7	1.0 (Reference)	1.0 (Reference)
After-hours	45357	79.2	11942	20.8	0.9 (0.9-1.0)	0.8 (0.8-0.8)
**Season of arrival**						
Spring	32582	78.2	9097	21.8	1.1 (1.0-1.1)	1.1 (1.1-1.2)
Summer	19607	78.8	5270	21.2	1.0 (1.0-1.0)	1.1 (1.1-1.2)
Autumn	22148	78.4	6094	21.6	1.0 (1.0-1.1)	1.1 (1.0-1.1)
Winter	34660	79.1	9167	20.9	1.0 (Reference)	1.0 (Reference)
**Top 5 conditions**						
Trauma vs. all others^c^	32154	87.7	4507	12.3	0.4 (0.4-0.5)	0.5 (0.5-0.6)
Infectious vs. all others^c^	17152	84.4	3168	15.6	0.7 (0.7-0.7)	0.7 (0.7-0.8)
Ear, nose & throat vs. all others^c^	11804	92.3	990	7.7	0.3 (0.3-0.3)	0.3 (0.3-0.4)
Gastrointestinal vs. all others^c^	8668	73.0	3201	27.0	1.5 (1.5-1.6)	1.5 (1.4-1.6)
Respiratory vs. all others^c^	5864	52.8	5239	47.2	4.1 (4.1-4.3)	2.6 (2.5-2.8)
*Missing*	*2551*	*49.7*	*2578*	*50.3*	*Not included*	

*ED* Emergency department, *SA* South Australia, *QLD* Queensland, *OR* Odds ratio, *CI* Confidence interval, *IQR* Inter-quartile range. Note. Presentations ending in a “did not wait”, “deceased” or “unknown” were excluded from regression analysis. The all-sites multivariate model contained data from all sites, but only contained half of the data from Sweden, due to a large number of records without a diagnosis.

^a^Triage category: emergency (within 10 minutes: Australasian Triage Scale, ATS 1 or 2, Swedish triage red or orange), urgent (within 60 minutes: ATS 3 or 4, Swedish triage yellow), less urgent (>60 minutes: ATS 5, Swedish triage green or blue);

^b^Time of arrival: In-hours: 06:00-17:59; After-hours: 18:00-05:59;

^c^reference category is all other diagnoses; Did not enter: tested, but not statistically significant after adjusting for other variables in model; Not tested: due to missing data at one or more sites.

In multivariate modelling, predictors of a more than two-fold higher risk of admission were high urgency triage categories, arrival by ambulance, presentation at children’s hospital, and respiratory diagnosis (Table 4).

At each site individually, common factors predictive of admission included: triage category, referral source, arrival by ambulance, presentation in summer vs. winter, and for the Australian sites, presentation with a respiratory or gastrointestinal condition (Table 5). The magnitude of effect of some of these predictors varied by site. For example, the OR of admission at the Swedish mixed ED when the child arrived by ambulance was 3.9 (95% CI 3.4–4.5) whereas at the mixed ED in QLD, Australia, it was 1.8 (95% CI 1.6–2.0). Triage urgency was a major predictor of hospital admission in all EDs, however this also varied by site. For example, the OR of admission at the Swedish mixed ED when the child was assigned an emergency triage category was 6.4 (95% CI 5.6–7.5) and at the children’s ED in QLD, it was 26.5 (95% CI 21.7–32.3).

**Table 5:** Predictors of admission for children: All sites and site-specific multivariate models, showing adjusted odds ratios and 95% confidence intervals

Predictor	All sites model	ED A, SA [Children's]	ED B, QLD [Children's]	ED C, QLD [Mixed]	ED D, SA [Mixed]	ED E, Sweden [Mixed]
		(*n* = 55,697)	(*n* = 34,657)	(*n* = 15,956)	(*n* = 16,677)	(*n* = 15,638)
**Age**						
< 12 months	0.9 (0.9–0.9)*	0.9 (0.9–1.0)	0.9 (0.8–1.0)	0.7 (0.6–0.8)*	1.3 (1.1–1.5)*	1.0 (0.9–1.2)
1	0.8 (0.8–0.9)*	0.9 (0.8–1.0)	0.8 (0.7–0.9)*	0.8 (0.7–0.9)*	1.0 (0.8–1.2)	0.7 (0.6–0.9)*
2–5	0.8 (0.8–0.9)*	0.9 (0.8–0.9)*	0.9 (0.8–1.0)	0.7 (0.6–0.8)*	0.9 (0.8–1.1)	0.8 (0.7–1.0)*
6–11	0.8 (0.8–0.9)*	0.8 (0.8–0.9)*	0.9 (0.9–1.0)	0.8 (0.7–0.9)*	0.7 (0.6–0.8)*	1.0 (0.9–1.2)
12–17	1.0 (reference)	1.0 (reference)	1.0 (reference)	1.0 (reference)	1.0 (reference)	1.0 (reference)
**Sex**						
Female	1.0 (reference)	did not enter	1.0 (reference)	did not enter	did not enter	did not enter
Male	1.0 (1.0–1.1)*		0.9 (0.9–1.0)*			
**Indigenous status**						
Not Aboriginal or Torres Strait Islander	Not tested	1.0 (reference)	did not enter	1.0 (reference)	did not enter	Not collected
Aboriginal or Torres Strait Islander		1.3 (1.2–1.5)*		1.4 (1.1–1.8)*		
**Language spoken at home**						
Not English	Not tested	Not tested	0.7 (0.6–0.9)*	0.5 (0.3–0.9)*	did not enter	Not collected
English			1.0 (reference)	1.0 (reference)		
**Insurance status**						
Public	Not tested	1.0 (reference)	did not enter	1.0 (reference)	did not enter	Not collected
Other		0.9 (0.9–1.0)*		1.2 (1.0–1.5)*		
**Referral source**						
Primary care	1.4 (1.3–1.5)*	1.5 (1.4–1.6)*	1.6 (1.4–1.7)*	1.2 (1.1–1.4)*	1.5 (1.3–1.8)*	2.3 (2.0–2.6)*
Self	1.0 (reference)	1.0 (reference)	1.0 (reference)	1.0 (reference)	1.0 (reference)	1.0 (reference)
**Mode of arrival**						
Ambulance	2.2 (2.1–2.3)*	3.6 (3.4–3.9)*	2.4 (2.3–2.6)*	1.8 (1.6–2.0)*	2.1 (1.8–2.4)*	3.9 (3.4–4.5)*
Private	1.0 (reference)	1.0 (reference)	1.0 (reference)	1.0 (reference)	1.0 (reference)	1.0 (reference)
**Triage category**^**a**^						
Emergency	20.8 (18.6–23.3)*	22.8 (19.0–27.2)*	26.5 (21.7–32.3)*	20.3 (8.9–46.1)*	11.8 (8.6–16.2)*	6.4 (5.6–7.5)*
Urgent	4.8 (4.3–5.3)*	4.0 (3.4–4.8)*	4.3 (3.5–5.1)*	5.2 (2.3–11.8)*	3.0 (2.3–4.1)*	2.1 (1.9–2.4)*
Less urgent	1.0 (reference)	1.0 (reference)	1.0 (reference)	1.0 (reference)	1.0 (reference)	1.0 (reference)
**Day type of arrival**						
Weekend	0.9 (0.9–0.9)*	0.9 (0.9–1.0)*	0.9 (0.8–0.9)*	0.9 (0.8–1.0)*	did not enter	did not enter
Weekday	1.0 (reference)	1.0 (reference)	1.0 (reference)	1.0 (reference)		
**Time of arrival**^b^						
Afterhours	0.8 (0.8–0.8)*	0.7 (0.7–0.7)*	did not enter	0.8 (0.7–0.9)*	did not enter	did not enter
In-hours	1.0 (reference)	1.0 (reference)		1.0 (reference)		
**Season of arrival**						
Spring	1.1 (1.1–1.2)*	1.2 (1.1–1.2)*	1.2 (1.2–1.3)*	1.0 (0.9–1.1)	1.1 (1.0–1.2)	1.0 (0.9–1.1)
Summer	1.1 (1.1–1.2)*	1.2 (1.1–1.3)*	1.1 (1.0–1.3)*	1.2 (1.0–1.3)*	1.3 (1.1–1.5)*	1.2 (1.0–1.3)*
Autumn	1.1 (1.1–1.2)*	1.2 (1.1–1.3)*	1.0 (0.9–1.1)	1.2 (1.1–1.4)*	1.2 (1.1–1.4)*	1.2 (1.0–1.3)*
Winter	1.0 (reference)	1.0 (reference)	1.0 (reference)	1.0 (reference)	1.0 (reference)	1.0 (reference)
**Top 5 conditions**						
Trauma vs. all others^c^	0.6 (0.6–0.6)*	0.5 (0.5–0.5)*	0.6 (0.6–0.7)*	0.5 (0.4–0.5)*	0.1 (0.1–0.2)*	Not tested
Infectious vs. all others^c^	0.8 (0.8–0.9)*	0.8 (0.8–0.9)*	0.7 (0.7–0.9)*	1.2 (1.0–1.4)*	0.6 (0.5–0.7)*	Not tested
Ear, nose & throat vs. all others^c^	0.4 (0.4–0.4)*	0.4 (0.3–0.4)*	0.3 (0.2–0.3)*	0.7 (0.6–0.9)*	0.3 (0.2–0.4)*	Not tested
Gastrointestinal vs. all others^c^	1.7 (1.6–1.8)*	1.7 (1.5–1.8)*	1.3 (1.2–1.5)*	1.8 (1.5–2.1)*	1.5 (1.3–1.8)*	Not tested
Respiratory vs. all others^c^	2.3 (2.1–2.4)*	2.4 (2.3–2.6)*	2.8 (2.5–3.1)*	3.3 (2.8–3.8)*	2.6 (2.2–2.9)*	Not tested

*ED* Emergency department, *SA* South Australia, *QLD* Queensland, *OR* Odds ratio, *CI* Confidence interval

^*^Statistically significant (*p* < 0.05), including if the confidence interval ended or began with 1.0;

^a^Triage category: Emergency (within 10 min: ATS 1 or 2, Swedish triage red or orange), Urgent (within 60 min: ATS 3 or 4, Swedish triage yellow), less urgent (> 60 min: ATS 5, Swedish triage green or blue)

^b^Time of arrival: In-hours: 06:00–17:59; After-hours: 18:00–05:59;

^c^reference category is all other diagnoses; Did not enter: tested, but not statistically significant after adjusting for other variables in model; Not tested: due to missing data at one or more sites

## Discussion

In this multi-site study of children presenting to ED certain characteristics were predictive of admission to hospital, regardless of hospital type or location. To our knowledge, this study is the first to describe higher admission rates for children presenting to a children’s only hospital ED compared to a mixed hospital ED. Whilst our study controlled for different age distributions, diagnostic categories, arrival modes, and urgency, it could be that the children presenting to the children’s only EDs were ‘sicker’ or more complex than those presenting to the mixed EDs. This finding is reflective of a US report revealing that children with complex medical conditions represented 33% of admissions to children’s hospitals but only 20% of admissions for children seen in mixed hospitals [27]. In Australia, children’s only EDs are located within tertiary level hospitals that are resourced to provide specialist care. Thus, our findings may reflect practices where attendance and or referral of children with more complex medical problems and severe trauma/illness to these EDs are more common. Differences in treatment regimens between paediatric emergency medicine clinicians and general clinicians [28] may also explain the varying admission rates seen. Thus, whether or not the threshold for admission, case complexity, or clinician specialisation is different in children’s hospitals compared to mixed hospitals merits further study.

Some predictors of admission varied by location. Comparing the Swedish mixed ED to the two Australian mixed EDs, the same variables were significantly associated with admission (i.e., referral from primary care, arrival by ambulance, and triage category). However, in Sweden, the magnitude of the effect was greater for the first two variables, suggesting that these types of presentations represented more acutely unwell children than in the Australian sites, or access to and utilisation of community based services is different in some way, despite health care (including use of ambulance services) for children being free in both countries. In the Australian sites, Aboriginal or Torres Strait Islander status was a significant predictor of admission at two of the four EDs. This could be because of the higher proportion of Aboriginal or Torres Strait Islander children living in the hospital catchment areas, potential varying accuracy of data capture between sites or varying practices and policies underpinning care delivery of Aboriginal or Torres Strait Islander children. Presentation after-hours resulted in a lower admission rate for children presenting to the two hospitals where this analysis was possible (SA children’s and QLD mixed). Whilst differences in admission rates by time of presentation may be associated with the availability of paediatric emergency specialists [29], access to primary care centres and general practitioners, this particular factor was not able to be comprehensively reported across all sites in our study.

Some factors predictive of hospital admission for children in our study have been noted elsewhere [15–20]. A summary of findings from one Irish study [18], four US studies [15, 16, 19, 20] and this study (all of which used paediatric populations and reported multivariate factors predictive of admission) is presented in Table 6. Ambulance arrival carried an increased likelihood of admission in all of our study sites and two US studies [16, 19]. Referral from primary care (i.e., general practitioner) was predictive of admission for children in this study, and in other US paediatric populations [20]. Triage category was amongst the most powerful of predictors of admission in our study and also predictive in other US and Irish studies [15, 16, 18]. The lower odds of admission during weekends and after-hours noted in the Australian sites of our study may suggest challenges with the admission process during these times. This is not necessarily unique to our study, with higher odds of admission on weekdays for children reported elsewhere [18]. Of possible concern here is the subsequent outcomes with children admitted on the weekend found in one US study to have significantly higher odds of unplanned readmission within 30 days of discharge compared to children admitted on weekdays [30]. Further research in this area is required in other countries. Some factors not collected in our study but reported elsewhere as predictive of hospital admission for children (such as distance travelled [17, 18] and vital signs [15, 16, 19, 20]) can be easily integrated into existing health data systems and further used to support the development of models for early hospital admission decision making.

**Table 6:** Predictors of hospital admission in children from multivariate modelling studies from Ireland, USA, Australia and Sweden

Predictor	**Country of Study**			
	Ireland [One study] [18]	USA [Four studies] [15, 16, 19, 20]	Australia [This study]	Sweden [This study]
**ED type (Children only or mixed children and adults)**	N/A (single site; children’s only ED)	N/A (single site studies) [15] Not reported (multi-site studies) [16, 19, 20]	Yes: children’s only	N/A (single site; mixed ED)
**Age**	EFM	Yes [19]^a^; younger (< 3 months) [15, 16, 20]	Yes: older child ^ED: A,B,C,D^	Yes: older child
**Sex**	EFM	EFM [15]	Yes: female ^ED: B^	EFM
**Arrival mode**	EFM	Yes: ambulance [16, 19] EFM [15]	Yes: ambulance ^ED: A,B,C,D^	Yes: ambulance
**Referral source**	Yes^a^	Yes: physician / other ED [20]	Yes: primary care ^ED: A,B,C,D^	Yes: primary care
**Triage category**	Yes^a^	Yes [15]^a^; higher acuity [16]	Yes: higher acuity ^ED: A,B,C,D^	Yes: higher acuity
**Presenting/chief complaint**	Yes^a^	Yes [15]^a^; altered mental status [16, 20], chronic disease, fever (neonate), pregnancy, psychological/behavioural [16], psychiatric reasons [19], abdominal pain (in adolescents), immunodeficiency [20]	Yes: Infectious ^ED: C^ Gastrointestinal ^ED: A,B,C,D^ Respiratory ^ED: A,B,C,D^	EFM
**Weekday presentation**	Yes		Yes ^ED: A,B,C^	EFM
**In-hours presentation**			Yes ^ED: A,C^	EFM
**Insurance status**			Yes: Private ^ED: C^	NC
**Race / Ethnicity**			Yes (Aboriginal / Torres Strait Islander) ^ED:A,C^	NC
**Language spoken at home**			Yes: English ^ED: B,C^	NC
**Distance travelled**	Yes^a^			
**Registration month**	Yes^a^			
**ED location**	Yes^a^			
**Previous admission**	Yes			
**Vital signs**		Yes: HR [16, 19], temperature [16], pulse oximetry [15, 19], respiratory rate [19], systolic BP [19, 20], diastolic BP [19, 20] EFM: GCS [15, 16], BP [15, 16], HR [15], pulse oximetry [16]		
**Currently taking prescription medicines**		Yes [16] Yes (for asthma) [20] EFM [15]		
**Past medical history**		Yes [15] EFM [16]		
**Abnormal laboratory values**		Yes: low serum bicarbonate, high potassium, blood urea nitrogen, white blood cell count [20]		
**Oxygen requirement in ED**		Yes [20]		
**Other**		Indwelling medical device [20]		

*ED* Emergency department, *HR* Heart rate, *BP* Blood pressure, *GCS* Glasgow Coma Scale, *EFM* Excluded from model: if variable considered in study, but not included in the final model, *NC* Data not collected for the Swedish site

^a^Studies noted the variable was predictive of admission, but did not specify further; presented in table are predictors of hospital admission where odds/risk noted (predictors with lower odds/risk, i.e., protective of admission, are not presented – refer to specific study); studies 18 and 19 report graded variables of importance (those noted here are their top 8 – refer to specific study for others)

### Limitations

Although the data sets in this study are from 2011–2012, some characteristics of the sample in the four Australian hospitals were broadly similar to national data available on children from 2021–22 [3]. We recognise that there have been some significant changes in paediatric emergency care delivery over the last decade. This includes tremendous growth [3, 6], and a pandemic which has altered presentation patterns [31–33] since the time of this study potentially impacting on the relevance of our findings. This research provides baseline evidence to inform if and how practice changes may have impacted hospital admission. This study used retrospective data where there is the potential for inaccuracies and a cause-and-effect relationship cannot be established. Considerable missing data was evident in some fields (e.g., language spoken at home, Indigenous Status) impacting on our ability to comprehensively predict hospital admission with these variables. Efforts to better understand and competently cater for a culturally and linguistically diverse patient population are warranted [34, 35]. Improving the collection of culturally and linguistically diverse related information would be one way to contribute towards such efforts. Our analysis considered all hospital admissions. In Australia and Sweden, ED short stay unit admissions and ward admissions tend to reflect different patient groups. Thus, care is required in interpreting our findings. We used forward stepwise regression to identify factors predictive of hospital admission. We acknowledge other approaches (e.g., penalised maximum likelihood estimation) [36] may be also used, however with large datasets and relatively frequent outcome of interest (as is the case in our study), standard and penalised models have been noted to perform similarly [37]. Although this was a multi-site study with data from four sites in Australia (from two different states) and one site from Sweden, we cannot generalise our findings to other sites with different profiles. Our findings do however provide a much more informed understanding of predictors of hospital admission for children that may be used to assist clinicians and hospital managers, supported by the use of artificial intelligence and machine learning algorithms. Such application has great potential to improve information use, especially in resource poor settings [38].

## Conclusions

Hospital admission rates for children varied based on hospital type (children’s only or mixed). Most factors predictive of hospital admission (triage category, referral from primary care, arrival by ambulance and older age) were consistent between sites. Children with certain diagnoses (especially pertaining to respiratory and gastrointestinal illnesses) were admitted in much higher proportions, regardless of age, triage category, location or type of hospital. Together, this information may be used to inform the development/enhancement of clinical pathways to potentially expedite admission processes for children.

### Supplementary Information

**Additional file 1: Table S1.** Variables and categorisations applied. **Table S2.** Variables available for modelling and the percentage of data missing by hospital.

## Data Availability

The datasets generated and/or analysed during the current study are not publicly available due to ethics and data privacy requirements.

## Abbreviations

EDs: Emergency departments
US: United States
UK: United Kingdom
QLD: Queensland
SA: South Australia
IQR: Inter-quartile range
CI: Confidence intervals
OR: Odds ratios
aOR: Adjusted odds ratios
