# Supplementary material for: Factors predictive of hospital admission for children via emergency departments in Australia and Sweden: an observational cross-sectional study
Source: BMC Health Serv Res. 2024 Feb 23;24:235. doi: 10.1186/s12913-023-09403-w (PMC10885502; doi:10.1186/s12913-023-09403-w)
Supplement: Supplementary file 1 — Additional file 1: Table S1. Variables and categorisations applied. Table S2. Variables available for modelling and the percentage of data missing by hospital. [file 12913_2023_9403_MOESM1_ESM.docx]

**Supplementary Material**

**Table S1. Variables and categorisations applied**

| **Variable** | **Categories** |
| --- | --- |
| Age (years and group) | Infancy (<12 months), toddler (1 year), early childhood (2-5 years), middle childhood (6-11 years) and early adolescence (12-17 years) [1] |
| Sex | male/female |
| Indigenous status (AUS sites only) | Aboriginal or Torres Strait Islander |
| Language (AUS sites only) | English, not English |
| Insurance (AUS sites only) | Public only, private insurance, no insurance |
| Referral source | Self-referred, primary health, community |
| Mode of arrival | Private transport, ambulance, other |
| Triage category (Aus)†: Australasian Triage Scale (ATS) [2] | 1 - Immediate  2 - Within 10 minutes coded as emergency  3- Within 30 minutes  4 - Within 60 minutes coded as urgent  5 - Within 120 minutes coded as less urgent |
| Triage category (Sweden) †: Rapid Emergency Triage and Treatment System-paediatrics (RETTS-p) [3] | Red - Immediate  Orange - Within 10 minutes coded as emergency  Yellow - Within 60 minutes coded as urgent  Green - Within 120 minutes  Blue - Within 240 minutes coded as less urgent |
| Diagnosis (based on International Classification of Diseases-10^th^ edition) [4] | The principal diagnosis code assigned at ED discharge was mapped to one of 23 major diagnostic categories. |
| Departure status | Home  Admitted  Did not wait  Deceased  Unknown |

†Note. For regression analysis, triage category was grouped as emergency (within 10 minutes, ATS 1 or 2, Swedish triage red or orange), urgent (within 60 minutes: ATS 3 or 4, Swedish triage yellow), and less urgent (>60 minutes: ATS 5, Swedish triage green or blue). For categories where <1% of entries were evident further collapsing was performed: a referral type of “community” was included in the “primary care” category; and arrival mode of “other” was combined with private transport.

**Table S2. Variables available for modelling and the percentage of data missing by hospital**

| Variable | **ED A, SA [Children's]** | **ED B, QLD [Children's]** | **ED C, QLD [Mixed]** | **ED D, SA [Mixed]** | **ED E, Sweden [Mixed]** | **All Sites** |
| --- | --- | --- | --- | --- | --- | --- |
| Age (years) |  |  |  |  |  |  |
| Sex (male/female) |  |  |  |  |  |  |
| Aboriginal or Torres Strait Islander |  |  |  |  |  |  |
| Language | 78.1 |  |  |  |  | 42.3 |
| Insurance |  |  |  |  |  | 11.1 |
| Referral source |  |  |  |  |  |  |
| Mode of arrival |  |  |  |  |  |  |
| Diagnosis |  | 7.1 | 5.9 | 10.5 | 50.0 | 9.1 |
| Triage category |  |  |  |  | 13.0 |  |
| Departure status |  |  |  |  |  |  |
|  |  |  |  |  |  |  |
| KEY |  |  |  |  |  |  |
|  | <5% missing |  |  |  |  |  |
| actual % missing | >5% but <20% missing | |  |  |  |  |
| actual % missing | >20% missing |  |  |  |  |  |
|  | Entirely missing |  |  |  |  |  |

**References for Supplementary Material**

1. Williams K, Thomson D, Seto A, et al. Standard 6: Age Groups for Pediatric Trials. Pediatrics. 2012; 129(Supp 3): S153-60.

2. Australasian College of Emergency Medicine. Guidelines on the implementation of the Australasian Triage Scale in emergency departments.

https://acem.org.au/getmedia/51dc74f7-9ff0-42ce-872a-0437f3db640a/G24_04_Guidelines_on_Implementation_of_ATS_Jul-16.aspx Accessed 20 Oct 2021.

3. Magnusson C, Herlitz J, Karlsson T, Axelsson C. Initial assessment, level of care and outcome among children who were seen by emergency medical services: a prospective observational study. Scand J Trauma Resusc Emerg Med. 2018; 26 (1): 88.

4. Independent Hospital Pricing Authority. Australian Refined Diagnosis Related Groups Version 6.x. IHPA 2013 <https://www.ihpa.gov.au/publications/ar-drg-version-60x> Accessed 20 Oct 2021.
